# Evodiamine inhibits RANKL‐induced osteoclastogenesis and prevents ovariectomy‐induced bone loss in mice

**DOI:** 10.1111/jcmm.13955

**Published:** 2018-11-19

**Authors:** Haiming Jin, Lingya Yao, Kai Chen, Yuhao Liu, Qingqing Wang, Ziyi Wang, Qian Liu, Zhen Cao, Jacob Kenny, Jennifer Tickner, Xiangyang Wang, Jiake Xu

**Affiliations:** ^1^ Department of Orthopaedic Surgery The Second Affiliated Hospital and Yuying Children's Hospital of Wenzhou Medical University Wenzhou China; ^2^ School of Biomedical Sciences The University of Western Australia Perth Western Australia; ^3^ The Lab of Orthopaedics and Traumatology of Lingnan Medical Research Center Guangzhou University of Chinese Medicine Guangzhou Guangdong China; ^4^ Research Centre for Regenerative Medicine and Guangxi Key Laboratory of Regenerative Medicine Guangxi Medical University Guangxi China; ^5^ Department of Biomedical Materials Science Third Military Medical University Chongqing China

**Keywords:** Ca^2+^ oscillation, evodiamine, NF‐κB, osteoclast, osteoporosis, ovariectomy

## Abstract

Postmenopausal osteoporosis (PMO) is a progressive bone disease characterized by the over‐production and activation of osteoclasts in elderly women. In our study, we investigated the anti‐osteoclastogenic effect of evodiamine (EVO) in vivo and in vitro, as well as the underlying mechanism. By using an in vitro bone marrow macrophage (BMM)‐derived osteoclast culture system, we found that EVO inhibited osteoclast formation, hydroxyapatite resorption and receptor activator of NF‐κB ligand (RANKL)‐induced osteoclast marker gene and protein expression. Mechanistically, we found that EVO inhibited the degradation and RANKL‐induced transcriptional activity of IκBα. RANKL‐induced Ca^2+^ oscillations were also abrogated by EVO. In vivo, an ovariectomized (OVX) mouse model was established to mimic PMO, and OVX mice received oral administration of either EVO (10 mg/kg) or saline every other day. We found that EVO can attenuate bone loss in OVX mice by inhibiting osteoclastogenesis. Taken together, our findings suggest that EVO suppresses RANKL‐induced osteoclastogenesis through NF‐κB and calcium signalling pathways and has potential value as a therapeutic agent for PMO.

## 1 INTRODUCTION

Bone is a dynamic organ that is moulded, shaped and repaired throughout life via osteoblast‐mediated bone formation and osteoclast‐mediated bone resorption.^1^ Postmenopausal osteoporosis (PMO) is a common bone disease in elderly women featured by an imbalance between the activities of osteoclasts and osteoblasts.^2^, ^3^ The enhanced number and function of osteoclasts, resulting from oestrogen deficiency, has been determined to be the key driver of the pathologic changes observed in PMO.^4^ Therefore, inhibition of osteoclast differentiation and function could be a promising therapeutic strategy for attenuating the progression of PMO.

Osteoclasts are multinucleated, specialized bone‐resorbing cells that are derived from the monocyte/macrophage lineage. The formation of osteoclasts, also called osteoclastogenesis, is a multi‐stage process regulated by a number of genetic, humoural and mechanical factors. Among these factors, macrophage colony‐stimulating factor (M‐CSF) and receptor activator of nuclear factor‐kappa B (NF‐κB) ligand (RANKL) are well‐known as the key cytokines in osteoclastogenesis.^5^, ^6^ The cytokine M‐CSF is a prerequisite for osteoclast precursor proliferation and survival, whereas RANKL, a tumour necrosis factor (TNF) family cytokine, controls the function and survival of mature osteoclasts through interaction with its receptor RANK.^1^ Following the binding of RANKL to the RANK receptor, multiple intracellular signalling events are activated, including NF‐κB and calcium signalling.^7^, ^8^, ^9^ Ultimately, these signal transduction pathways lead to the expression and activation of transcription factors such as nuclear factor of activated T cells‐c1 (NFATc1) and activator protein‐1 (AP‐1), both of which are crucial for the differentiation of osteoclast precursors.^9^, ^10^, ^11^ Thus, blocking the intracellular signalling stimulated by RANKL is considered a major therapeutic target for the treatment of PMO.

Evodiamine (EVO) is an alkaloidal compound extracted and purified from the unripe fruit of Evodia rutaecarpa, a type of traditional Chinese medicine (TCM) with a long history.^12^ EVO has been reported to possess multiple pharmacological activities, including antimicrobial,^13^ anti‐tumour^14^ and anti‐inflammatory effects.^15^, ^16^ A previous study reported that EVO significantly suppresses zymosan‐induced inflammation by inhibiting the NF‐κB signalling pathway in the murine RAW264.7 macrophage cell line.^16^ EVO also exerts neuroprotective effects via down‐regulated NF‐κB expression to protect against permanent middle cerebral artery occlusion‐induced brain injury in mice.^17^ However, the effect of EVO on osteoclastogenesis remains unknown. Therefore, in our study, we investigated the inhibitory effects of EVO on osteoclast differentiation and function, as well as the underlying mechanism of EVO on RANKL‐treated osteoclasts. Furthermore, an ovariectomy (OVX)‐induced bone loss mouse model was established to mimic PMO, and the protective role of EVO against bone loss in PMO was examined.

## 2 MATERIALS AND METHODS

### 2.1 Ethics statement

The study was approved by the Animal Care and Use Committee at the Wenzhou Medical College. All surgical interventions, treatments and post‐operative animal care procedures were performed in accordance with the National Institutes of Health (NIH) Guide for the Care and Use of Laboratory Animals.

### 2.2 Reagents and antibodies

EVO (purity >98%) was purchased from Nantong Feiyu Biological Technology Co, Ltd. (Nantong, China). EVO was dissolved in DMSO as a 20‐mmol/L stock solution and stored at −20°C. Further dilution was performed in cell culture medium. Primary antibodies against NFATc1, c‐Fos, integrin‐β3, CTSK and β‐actin were obtained from Santa Cruz Biotechnology (San Jose, CA). Primary antibodies against IκBα, p65 and phospho‐p65 were obtained from Cell Signaling Technologies (Beverly, MA, USA). A V‐ATPase d2 antibody was generated as previously described.^18^ An MTS assay kit and a luciferase assay system were purchased from Promega (Madison, WI, USA). A leukocyte acid phosphatase staining kit was purchased from Sigma‐Aldrich (Sydney, Australia). Recombinant macrophage colony‐stimulating factor (M‐CSF) was obtained from R&D Systems (Minneapolis, MN). Recombinant GST‐rRANKL protein was synthesized and purified as previously described.^19^ The cell culture medium, alpha‐modified minimal essential medium (α‐MEM) and foetal bovine serum (FBS) were purchased from Thermo Fisher Scientific (Scoresby, Vic., Australia).

### 2.3 Cell culture

Bone marrow macrophages (BMMs) were isolated from the bone marrow of 6‐week‐old C57BL/6 mice, which were euthanized according to the procedures approved by the Animal Ethics Committee of the University of Western Australia (RA/3/100/1244). The extracted cells were collected by centrifugation, cultured in a 75 cm^2^ culture flask with α‐MEM (adding 10% FBS, a 1% antibiotic mixture of penicillin and streptomycin and 50 ng/mL M‐CSF) and incubated in an atmosphere of 5% CO_2_ at 37°C. The culture medium was changed every 2 days, and cells were passaged when 90%‐100% confluence were attained. BMMs from passages one to three were used in this study.

### 2.4 Cell viability assay

Cell viability was determined using an MTS (3‐(4,5‐dimethylthiazol‐2‐yl)‐2,5‐diphenyl tetrazolium bromide) assay following the manufacturer's protocol. Passage 1‐3 BMMs were seeded into 96‐well plates (5 × 10^3^ cell/well) and incubated with 50 ng/mL of M‐CSF at 37°C for 24 hours. Then the cells were treated with various concentrations of EVO (1, 2.5, 5, 10 and 20 μmol/L). After 48 hours of treatment, the cells were washed with PBS, 100 μL of non‐FBS medium (α‐MEM) containing 20 μL of MTS solution was added to each well, and the plate was incubated for an additional 2 hours. The absorbance of the wells was then measured at 490 nm by a micro‐plate reader (ThermoFisher, Waltham, MA, USA).

### 2.5 In vitro osteoclastogenesis assay

BMMs were plated at a density of 5 × 10^3^ cell/well in 96‐well plates and treated in complete medium containing M‐CSF and RANKL (50 ng/mL) with or without different concentrations of EVO (at 1, 2.5, 5 and 10 μmol/L). The EVO and medium were replaced every 2 days. After 5 days, cultured cells were fixed with 0.25% glutaraldehyde for 20 minutes at room temperature and then washed four times with PBS. Tartrate‐resistant acid phosphatase (TRAcP) enzymatic activity of the cells was detected using a leukocyte acid phosphatase staining kit according to the manufacturer's procedures. The number of TRAcP‐positive multinucleated cells (>3 nuclei) was counted under a light microscope (Nikon Corporation, Tokyo, Japan). Furthermore, the inhibitory effect of EVO (10 μmol/L) at different stages of osteoclast differentiation was also investigated. We examined the effects of EVO on RANKL‐induced osteoclast differentiation by treating at different time points from day 1 to day 5 after RANKL stimulation as follows: early stage: EVO was added on day 1 and removed on day 3; middle stage: EVO was added on day 3 and removed on day 5; late stage: EVO was added on day 5 and removed on day 6; and whole stage: EVO was added with RANKL as normal. Finally, cells were fixed and stained for TRAcP as described above.

### 2.6 Actin ring formation assay

BMMs were seeded into 96‐well plates and treated with different concentrations of EVO in the presence of 50 ng/mL M‐CSF and 50 ng/mL RANKL, as described above. After 5 days, paraformaldehyde (4%) was used to fix cells for 15 minutes at room temperature. After being washed with PBS three times, cells were permeabilized with 0.25% Triton X‐100 and then blocked with 3% BSA in PBS. Next, the F‐actin rings were stained with rhodamine‐conjugated phalloidin (Eugene, OR, USA), and the cell nuclei were stained with DAPI. Images were acquired using confocal laser scanning microscopy (Nikon, Tokyo, Japan). The number of multinucleated cells (>3 nuclei) and the number of nuclei were calculated.

### 2.7 Resorption pit assay

A resorption pit assay was used to evaluate osteoclast function. BMMs were seeded at a density of 8 × 10^4^ cell/well into 6‐well collagen‐coated plates and stimulated with M‐CSF (50 ng/mL) and RANKL (50 ng/mL) until mature osteoclasts formed. Cells were gently detached from the wells using a cell dissociation solution (Sigma, St. Louis, MO, USA) and then plated into hydroxyapatite‐coated 96‐well plates (Corning, New York, NY, USA) and bone slices in equal numbers. The mature osteoclasts were treated with different concentrations of EVO in the presence of M‐CSF (50 ng/mL) and RANKL (50 ng/mL). After 48 hours, half of the hydroxyapatite‐coated wells were bleached for 10 minutes to remove the cells and dried for hydroxyapatite resorption visualization using a light microscope, while the remaining hydroxyapatite‐coated wells were fixed and stained for TRAcP activity as described above to assess the number of multinucleated cells. Additionally, bone slices were stained with haematoxylin to detect resorption pits. Image J software (NIH, Bethesda, MD, USA) was used to analyse the percentage of hydroxyapatite resorption areas.

### 2.8 Luciferase reporter gene assays

The transcriptional activities of NF‐κB and NFATc1 were measured by luciferase reporter gene assays. RAW264.7 cells were stably transfected with either an NF‐κB‐responsive luciferase construct or an NFATc1‐responsive luciferase reporter construct.^20^, ^21^ The cells were seeded in 48‐well plates (1.5 × 10^5^ cells/well for NF‐κB and 5 × 10^4^ cells/well for NFATc1). The next day, the cells were pre‐treated with or without various concentrations of EVO for 1 hours, and then stimulated by 100 ng/mL RANKL (6 hours for NF‐κB and 24 hours for NFATc1). Analysis of luciferase activity was performed following the manufacturer's instructions (Promega, Sydney, Australia).

### 2.9 Intracellular Ca^2+^ measurement

To determine whether EVO can inhibit calcium signalling in the progression of osteoclast differentiation, we performed a measurement of intracellular Ca^2+^ oscillation as previously described. Briefly, BMMs were seeded into 48‐well plates (1 × 10^4^ cell/well) with M‐CSF (50 ng/mL) for 24 hours; next, the cells were treated with or without EVO (10 μmol/L) and RANKL (50 ng/mL) in the presence of M‐CSF (50 ng/mL) for another 24 hours. To label the intracellular free calcium, the cells were loaded with 4 μmol/L (100 μL/well) Fluo4 staining solution (Fluo4‐AM dissolved in 20% Pluronic F127 (w/v) in DMSO diluted in assay buffer) for 45 minutes after washing with assay buffer (Hanks’ balanced salt solution supplemented with 1 mmol/L probenecid and 1% FBS). The intracellular free calcium was detected at 488 nm by an inverted fluorescence microscope (Nikon Ti‐U). Then, the results were recorded and analysed by Nikon Basic Research Software. The images were scanned and plotted with an interval of 2 seconds for 3 minutes. A cell was considered as oscillating when two or more peaks were recorded. In addition, the differences between the maximum and minimum fluorescence intensities within the oscillating cell area in different groups were measured.

### 2.10 Real‐time PCR

Quantitative real‐time polymerase chain reaction (qRT‐PCR) was used to quantify the mRNA expression of TRAcP (Acp5), MMP9, CTSK and V‐ATPase d2. The total RNA of BMMs treated with or without different concentrations of EVO (5, 10 μmol/L) in the presence of M‐CSF (50 ng/mL) and RANKL (50 ng/mL) were extracted in 6‐well plates using TRIzol reagent (ThermoFisher Scientific, Scoresby, Australia). Next, 1000 ng of total RNA was reverse transcribed to synthesize cDNA using an RT‐PCR kit (Invitrogen, Carlsbad, CA, USA). The parameters of RT‐PCR were 5 minutes at 94°C, followed by 30 cycles of 40 seconds at 94°C, 40 seconds at 60°C and 40 seconds at 72°C, and then a final extension step of 5 minutes at 72°C. The reaction was performed using a ViiA^™^ 7 Real‐time PCR machine (Applied Biosystems, Paisley, UK). The cycle threshold (Ct) values were collected and normalized to the level of HPRT. Data were analysed using the 2^−ΔΔCT^ method. The specific primers used are listed in Table 1.

**Table 1:** Primer sequences used in qRT‐PCR

Gene	Forward primer	Reverse primer
CTSK	5′‐GGGAGAAAAACCTGAAGC‐3′	5′‐ATTCTGGGGACTCAGAGC‐3′
MMP‐9	5′‐CGTGTCTGGAGATTCGACTTGA‐3′	5′‐TTGGAAACTCACACGCCAGA‐3′
TRAcP	5′‐TGTGGCCATCTTTATGCT ‐3′	5′‐GTCATTTCTTTGGGGCTT ‐3′
c‐Fos	5′‐GCGAGCAACTGAGAAGAC‐3′	5′‐TTGAAACCCGAGAACATC‐3′
HPRT	5′‐GTTGGGCTTACCTCACTGCT‐3′	5′‐TAATCACGACGCTGGGACTG‐3′

### 2.11 Western blotting

Western blotting was performed using routine protocols. Treated BMMs were isolated using radioimmunoprecipitation assay (RIPA) lysis buffer (Millipore, Billerica, MA, USA) on ice. Equal amounts of proteins were separated by sodium dodecyl sulfate‐polyacrylamide gel electrophoresis (SDS‐PAGE) and transferred to polyvinylidene fluoride (PVDF) membranes (GE Healthcare, Silverwater, Australia). Membranes were blocked with 5% skim milk at room temperature for 2 hours and then incubated overnight at 4°C with one of the following primary antibodies: NFATc1, c‐Fos, integrin‐β3, CTSK, V‐ATPase d2, β‐actin, IκBα, p‐p65 and p65. Subsequently, the membranes were incubated with the corresponding secondary antibodies for 2 hours at room temperature. Protein bands were then visualized using an enhanced chemiluminescence (ECL) system (Amersham Pharmacia Biotech, Sydney, Australia).

### 2.12 Cell culture and assays of osteoblasts

To determine the effect of EVO on the differentiation and mineralization of osteoblasts, primary osteoblasts were isolated from the calvariae of neonatal mice by digestion with 0.1% collagenase and 0.2% dispase. The use of mice was conducted in accordance with an ethic approval RA/3/100/1244 by the Animal Ethics Committee of the University of Western Australia. Cells from passages 1‐2 were used for the present study. α‐MEM media with 10% FBS, 1% antibiotic mixture of penicillin and streptomycin, 10^−8^ mmol/L dexamethasone (DEX), 50 μg/mL ascorbic acid (A.A) and 10 mmol/L β‐glycerophosphate (β‐Gly) were used to stimulate the differentiation of osteoblasts. Meanwhile, 5 and 10 μmol/L EVO were added to the medium during osteoblastic differentiation. Alkaline phosphatase (ALP) assay, an early differentiation marker of osteoblasts, and alizarin red staining assay were performed using an ALP staining kit (Promega, WI, USA) and alizarin red staining kit (Sigma, St. Louis, MO, USA) separately on days 7 and 21 of culture according to the manufacturers’ suggested protocols.

### 2.13 Animal experiments

Seven‐week‐old female C57BL/6 mice were purchased from the Animal Center of the Chinese Academy of Sciences, Shanghai, China, and randomly divided into three groups (sham group, OVX group and OVX + EVO group, n = 10). All the mice were anaesthetized intraperitoneally with 2% (w/v) pentobarbital (40 mg/kg) and monitored by an assistant during the surgery. The bilateral OVX procedures were performed in the OVX group and OVX + EVO group using a dorsal approach. After the surgery, the animals were allowed to recover from surgery for 1 week prior to the experiments. The OVX + EVO group received EVO (10 mg/kg) dissolved in CMC by intragastric administration every other day for eight consecutive weeks. Mice in the sham group and OVX group were administered an equivalent volume of CMC. All animals were sacrificed 9 weeks after surgery, and femur bone samples were collected for micro‐CT scanning and histological examination.

### 2.14 Micro‐CT scanning

The structural properties of the trabecular bone of the distal femur were determined by high‐resolution micro‐CT (Skyscan 1176; Bruker, Belgium). The trabecular bone within the distal femur metaphysis was scanned at a 50‐kV tube voltage and 500‐μA current. Next, the three‐dimensional (3D) structure of the distal femur was reconstructed using Mimics 18.0 software (Materialise, Leuven, Belgium). Trabecular morphometry was characterized by measuring the bone volume per tissue volume (BV/TV), trabecular number (Tb.N), trabecular thickness (Tb.Th) and trabecular separation (Tb.Sp).

### 2.15 Histological examination

For haematoxylin and eosin (H&E) and TRAcP staining, the femurs of mice were excised, fixed in 4% formaldehyde at room temperature and decalcified in 10% ethylenediaminetetraacetic acid (EDTA). Then, femur bone specimens were paraffin‐embedded and sectioned to 5‐mm thickness with a microtome. Slides of each femur were stained with H&E. TRAcP staining was used to measure osteoclast activity in the bone tissue. All stained sections were scanned by the Aperio Scanscope, and bone histomorphometric parameters, including the BV/TV, osteoclast surface/bone surface (Oc.S/BS) and osteoclast number/bone surface (N.Oc/BS), were analysed by Bioquant Osteo software (Bioquant Image Analysis Corporation, Nashville, TN, USA).

### 2.16 Statistical analysis

The experiments were performed at least three times. The data obtained are expressed as the mean ± standard error of the mean (SEM). Statistical analysis was performed using GraphPad Prism version 5.0 software (GraphPad Software, San Diego, CA, USA). Inter‐group comparisons were performed using one‐way ANOVA followed by the Tukey test. Probability values of *P* < 0.05 were considered statistically significant.

## 3 RESULTS

### 3.1 Effects of EVO on BMM viability

**Figure 1:**
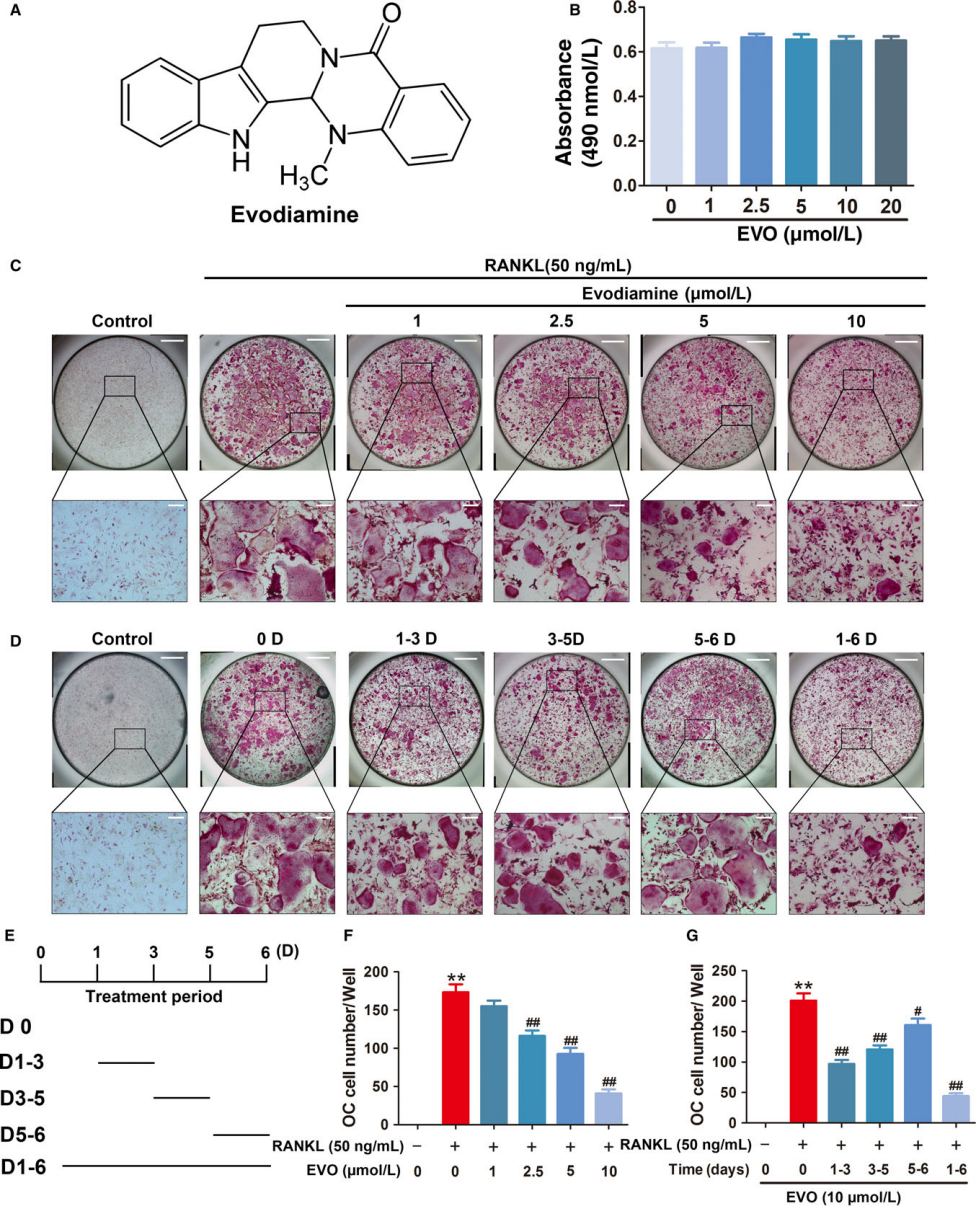
EVO inhibits RANKL‐induced osteoclastogenesis in vitro. (A) Chemical structure of EVO. (B) Effect of the indicated concentrations EVO on viability of BMMs for 48 hours, as measured by an MTS assay. (C) Representative TRAcP staining images of RANKL‐induced osteoclast formation treated with different concentrations of EVO for 5 days. Scale bar, 400 μm. Scale bar in the enlarged images, 100 μm. (D) Representative TRAcP‐staining images of RANKL‐induced osteoclast formation treated with 10 μmol/L EVO on the indicated days. (E) Schematic diagram of the EVO treatment time. (F, G) Quantification of TRAP ^+^ multinucleated cells (nuclei >3) shown in C and D. Data are presented as the mean ± SEM, *P < 0.05, **P < 0.01 relative to the control group. ^#^ *P* < 0.05, ^##^ *P* < 0.01 relative to the RANKL‐induced group. n = 3

The chemical structure of EVO is shown in Figure 1A. The cytotoxic effect of EVO (1, 2.5, 5, 10 and 20 μmol/L) on BMMs was assessed after 48 hours treatment using an MTS assay kit (Figure 1B). No significant cytotoxicity of EVO was observed at the concentrations used. Indeed, the results also indicated that EVO had no inhibitory effect on the M‐CSF‐induced proliferation of BMMs.

### 3.2 EVO inhibits RANKL‐induced osteoclastogenesis in vitro

To assess the effect of EVO on RANKL‐induced osteoclastogenesis, BMMs were treated with different concentrations of EVO in the presence of M‐CSF and RANKL. According to the results of TRAcP staining, stimulation by RANKL effectively induced osteoclast differentiation and formation, while increasing concentrations of EVO dose‐dependently inhibited the formation of TRAcP‐positive multinucleated osteoclasts (>3 nuclei) (Figure 1C, F). To determine the stage at which EVO inhibited osteoclast differentiation predominantly, cells were treated with EVO at different time phases. We found that EVO strongly inhibited RANKL‐induced osteoclast differentiation during the early and middle stages (Figure 1D, G). These results suggest that EVO acts mainly during the early and middle stages of osteoclast differentiation.

### 3.3 EVO inhibits RANKL‐induced F‐actin ring formation in osteoclasts

**Figure 2:**
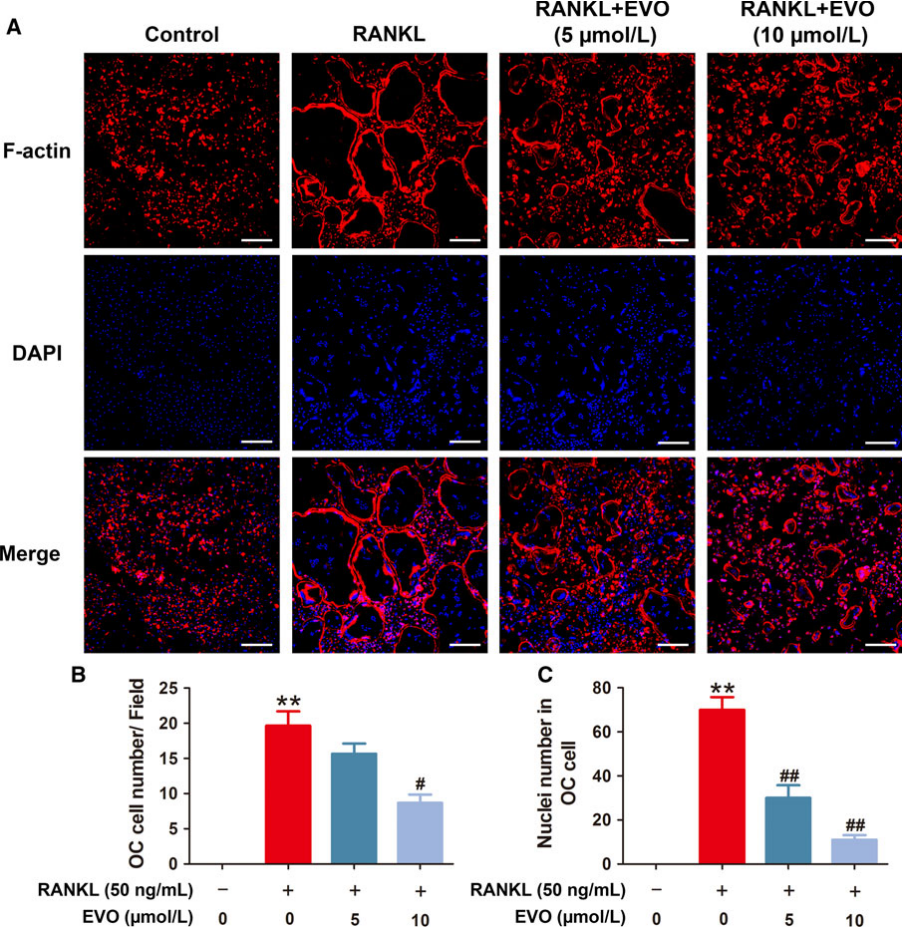
EVO inhibits RANKL‐induced F‐actin ring formation in osteoclasts. (A) The F‐actin ring formation was detected by the immunofluorescence combined with DAPI staining for nuclei. Scale bar, 200 μm. (B) Quantification of the osteoclasts treated with the indicated concentrations of EVO. (C) Average nuclei number per osteoclast under the different treatments. Data are presented as the mean ± SEM, *P < 0.05, **P < 0.01 relative to the control group. ^#^ *P* < 0.05, ^##^ *P* < 0.01 relative to the RANKL‐induced group. n = 3

Fibrous actin (F‐actin) rings are dynamic and the characteristic cytoskeletal structures of osteoclasts. A complete and large F‐actin structure is essential for osteoclasts involved in bone resorption. By staining with rhodamine‐conjugated phalloidin, osteoclasts with well‐defined podosome belts and multiple intact nuclei were observed upon stimulation by RANKL, whereas fewer and smaller osteoclasts with fewer nuclei were detected under EVO treatment (Figure 2). These results suggest that EVO suppresses RANKL‐induced formation of the F‐actin ring in osteoclasts.

### 3.4 EVO suppresses bone resorption by osteoclasts

**Figure 3:**
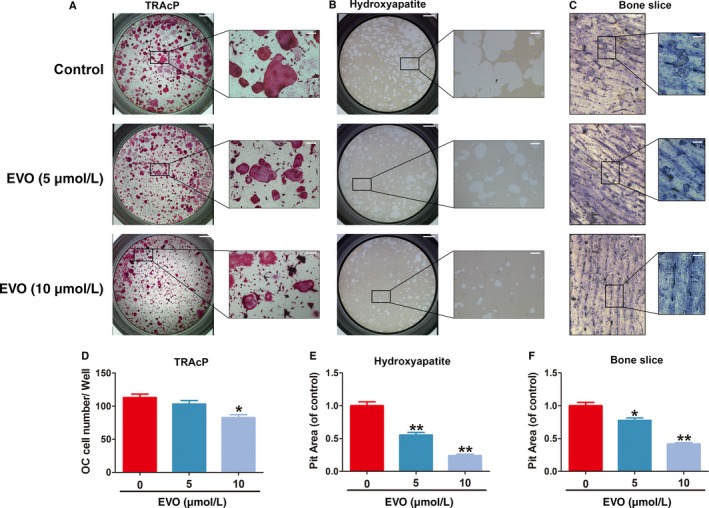
EVO suppresses bone resorption by osteoclasts. (A) Representative images of TRAcP‐stained osteoclasts on hydroxyapatite‐coated plates. Scale bar, 400 μm. Scale bar in the enlarged images, 100 μm. (B) Representative images of resorbing pits in hydroxyapatite‐coated wells. Scale bar, 400 μm. Scale bar in the enlarged images, 100 μm. (C) Representative images of resorbing pits in bones slice. Scale bar, 200 μm. Scale bar in the enlarged images, 100 μm. (D‐F) Quantification of the osteoclast number per well and resorbed area proportion under the different treatments. Data are presented as the mean ± SEM, *P < 0.05, **P < 0.01 relative to the control group. n = 3

To further confirm whether EVO inhibited the bone resorptive function of osteoclasts, BMM‐derived mature osteoclasts were seeded onto hydroxyapatite‐coated plates and bone slices and then treated with or without EVO for 48 hours. The results of a pit formation assay showed that the percentage of resorption pits per unit area on the surfaces of the hydroxyapatite‐coated plates and bone slices was smaller under EVO treatment than under control conditions (Figure 3). Thus, EVO attenuated the bone resorption activity of the mature osteoclasts.

### 3.5 EVO down‐regulates osteoclastogenic gene expression

**Figure 4:**
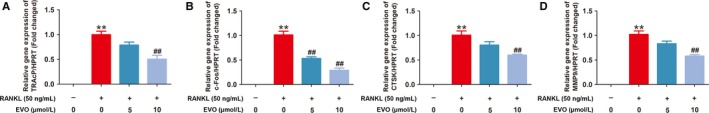
EVO down‐regulates osteoclastogenic gene expression. (A‐D) Real‐time PCR analysis was performed to detect the expression of osteoclastogenic genes c‐Fos, TRAcP (Acp5), MMP9 and CTSK. The expression levels of these genes were normalized to the expression of HPRT. Data are presented as the mean ± SEM, *P < 0.05, **P < 0.01 relative to the control group. ^#^ *P* < 0.05, ^##^ *P* < 0.01 relative to the RANKL‐induced group. n = 3

We next determined the effect of EVO on osteoclastogenic gene expression by real‐time PCR analysis, which is necessary for osteoclast formation and bone resorption. BMMs were treated with RANKL and M‐CSF for 5 days with or without different dosages of EVO. EVO significantly inhibited the RANKL‐induced up‐regulation of c‐Fos, TRAcP (Acp5), MMP9 and CTSK gene expression in a dose‐dependent manner (Figure 4).

### 3.6 EVO suppresses NFATc1 activity and the related protein expression

**Figure 5:**
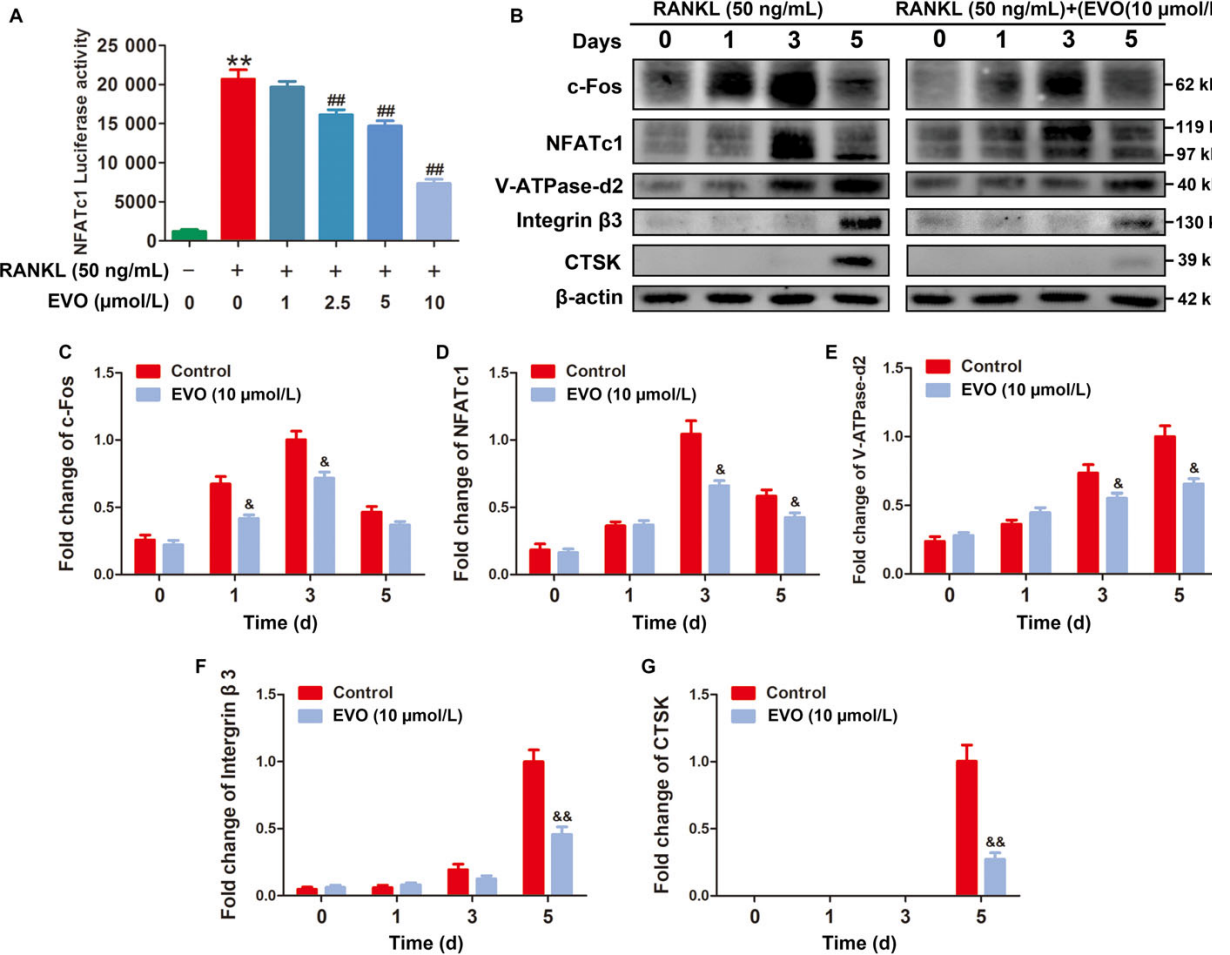
EVO suppresses NFATc1 activity and the related protein expression. (A) Luciferase reporter gene assay showing that EVO suppresses the RANKL‐induced transcriptional activity of NFATc1. (B) Representative images of western blots demonstrating the effect of EVO on the expression of NFATc1 and related proteins, including c‐Fos, CTSK, V‐ATPase d2 and integrin‐β3, induced by RANKL on the indicated days. Data are presented as the mean ± SEM, *P < 0.05, **P < 0.01 relative to the control group, ^#^ *P* < 0.05, ^##^ *P* < 0.01 relative to the RANKL‐induced group. ^&^ *P* < 0.05, ^&&^ *P* < 0.01 relative to the RANKL‐induced group on the same indicated day. n = 3

Whereas RANKL is considered the key osteoclastogenic cytokine, NFATc1 seems to be the master of osteoclastogenic transcription factors. To determine the RANKL‐induced NFATc1 transcriptional activity in the presence of EVO, luciferase reporter gene assays were performed. The results showed that EVO significantly inhibited the increased transcriptional activity of NFATc1 induced by RANKL (Figure 5A). Consistent with this result, the up‐regulated protein level of NFATc1 induced by RANKL was reversed by treatment with EVO during osteoclast differentiation. In addition, the expression of the protein related to NFATc1 was also detected in our study. c‐Fos, a member of the activator protein‐1 (AP‐1) transcription factor family, was suppressed by EVO treatment. The expression of downstream proteins of NFATc1, including CTSK, V‐ATPase d2 and integrin‐β3, was also inhibited by EVO treatment on day 3 and day 5 (Figure 5B‐G).

### 3.7 EVO inhibits RANKL‐induced NF‐κB signalling pathway and Ca^2+^ oscillation

**Figure 6:**
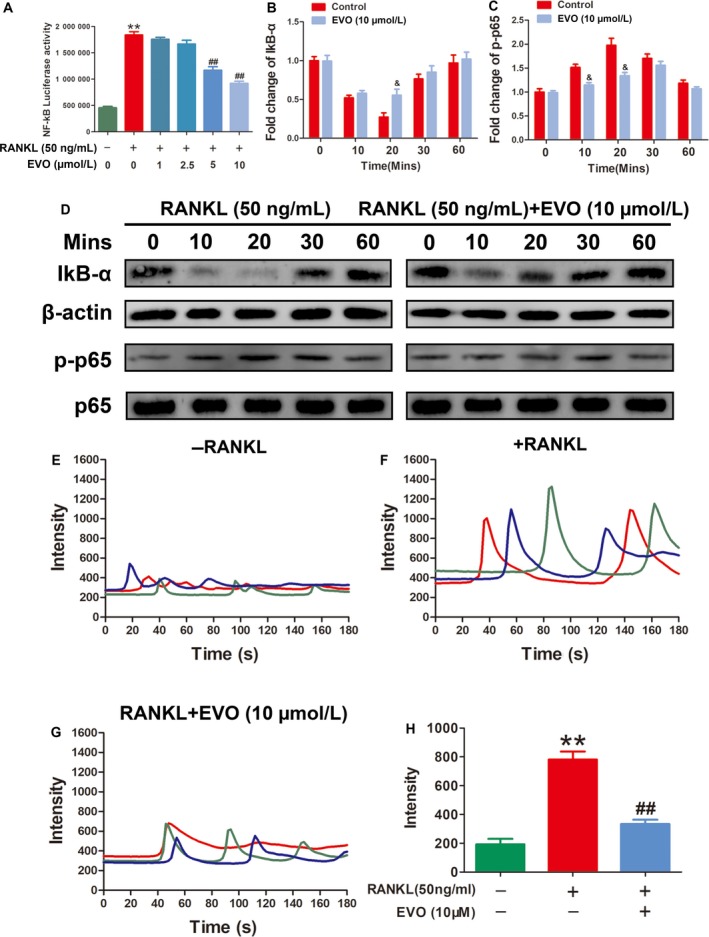
EVO inhibits the RANKL‐induced NF‐κB signalling pathway and Ca^2+^ oscillation. (A) Luciferase reporter gene assay showing that EVO suppresses the RANKL‐induced transcriptional activity of NF‐κB. (B, C) Ratios of the intensity of IκBα relative to that of β‐actin and of p‐p65 relative to that of p65. (D) Representative image of a western blot demonstrating the effect of EVO on IκBα degradation and phosphorylation of p65 induced by RANKL at the indicated times. (F‐G) Representative images of Ca^2+^ oscillation under the different treatments. (H) Quantitative analysis showing that EVO attenuated RANKL‐induced calcium oscillation. Data are presented as the mean ± SEM, *P < 0.05, **P < 0.01 relative to the control group, ^#^ *P* < 0.05, ^##^ *P* < 0.01 relative to the RANKL‐induced group. ^&^ *P* < 0.05, ^&&^ *P* < 0.01 relative to the RANKL‐induced group at the same indicated time. n = 3

Intracellular RANK signalling by its interaction with RANKL induces the activation of NF‐κB and Ca^2+^ oscillation, which subsequently mediate NFATc1 activation. NF‐κB is a protein complex composed of seven transcription factors and is normally located in the cytoplasm bound to its inhibitor IκB. To determine the effect of EVO on the RANKL‐induced NF‐κB signalling pathway, we detected the protein expression of IκB and the phosphorylation of NF‐κB p65 (Figure 6B‐D). The results showed that EVO (10 μmol/L) significantly inhibited the degradation of IκBα and the phosphorylation of p65 induced by RANKL. In addition, the transcriptional activity of NF‐κB was also assessed by luciferase reporter gene assays. NF‐κB transcriptional activity was markedly promoted by RANKL stimulation but decreased by EVO treatment (Figure 6A). Furthermore, we found that EVO significantly suppressed RANKL‐induced Ca^2+^ oscillation, indicating that calcium signalling was also involved in the inhibitory effect of EVO (Figure 6E‐H).

### 3.8 EVO did not affect the differentiation and mineralization of osteoblasts in vitro

Bone remodelling and homeostasis are maintained through a balance between bone resorption by osteoclasts and bone formation by osteoblasts.^1^ To determine the effect of EVO on the differentiation of osteoblasts, an ALP assay and alizarin red staining assay were performed. The results showed that EVO had no significant effect on osteoblast differentiation or mineralization compared with the control group (Figure S1A, B). Additionally, the toxicity of EVO on osteoblasts was evaluated. The results of the MTS assay revealed that the viability of osteoblasts was not affected by EVO at concentrations of 20 μmol/L and lower (Figure S1C). Taken together, these data indicated that EVO had no effect on the differentiation and mineralization of osteoblasts.

### 3.9 Effect of EVO on bone loss in OVX mice

**Figure 7:**
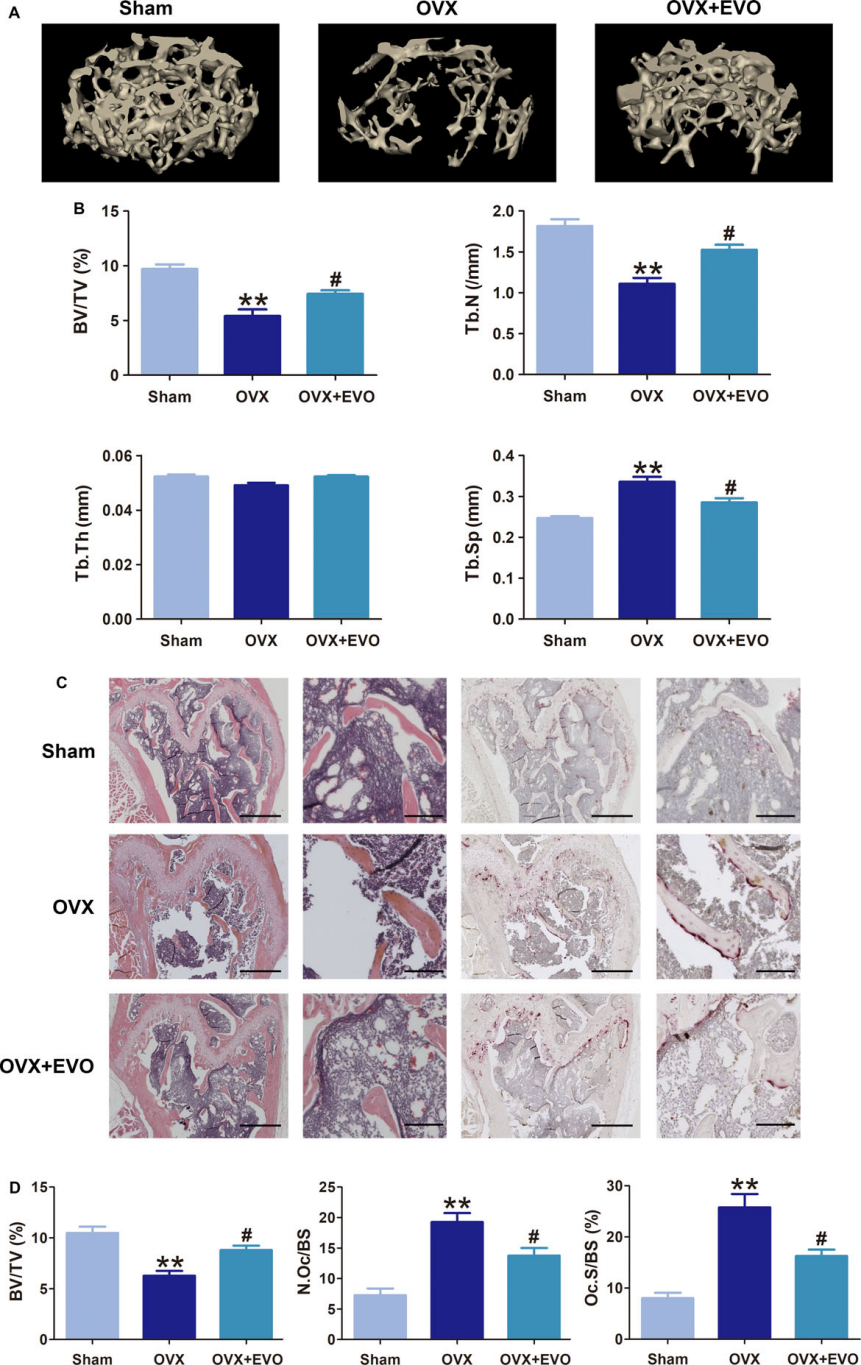
EVO prevents bone loss in OVX mice. (A) Representative 3D reconstruction micro‐CT images of the bone micro‐architecture in the different groups. (B) Bone volume per tissue volume (BV/TV), trabecular number (Tb.N), trabecular thickness (Tb.Th) and trabecular separation (Tb.Sp) were analysed with micro‐CT Skyscan CTAn software. (C) Representative H&E and ALP staining images of distal femurs in different groups. Scale bar, 500 μm. Scale bar in the enlarged images, 200 μm. (D) The BV/TV, osteoclast surface/bone surface (Oc.S/BS) and osteoclast number/bone surface (N.Oc/BS) ratios were analysed with BIOQUANT OSTEO software. Data are presented as the mean ± SEM, *P < 0.05, **P < 0.01 relative to the sham group. ^#^ *P* < 0.05, ^##^ *P* < 0.01 relative to the OVX group. n = 6

To study the potential therapeutic benefits of EVO on bone loss in vivo, an OVX mouse model was established to mimic PMO. Micro‐CT scanning and 3D reconstruction were performed to assess changes in the bone micro‐architecture in OVX mice, which revealed a significantly decreased trabecular bone mass in OVX mice compared to that in sham‐operated mice. However, compared to vehicle treatment, oral administration of EVO strongly attenuated the bone loss following ovariectomy, with significant increases in BV/TV and Tb.N and a decrease in Tb.Sp. Tb.Th was uniform across all groups in our study (Figure 7A, B). Meanwhile, these results were further supported by histological assessment of decalcified distal femoral sections stained for H&E and TRAcP. In H&E images, the value of BV/TV in the OVX + EVO group was significantly enhanced compared to that in the OVX group. In addition, the increased values of Oc.S/BS and N.Oc/BS in the femoral metaphysis induced by OVX were also markedly reduced by EVO treatment (Figure 7C, D). Thus, these data indicate that EVO can attenuate bone loss and micro‐architecture deterioration in the OVX mouse model.

## 4 DISCUSSION

Oestrogen deficiency in the elderly women is known to increase bone turnover, leading to increased formation and activation of osteoclasts.^4^ Progressive and excessive bone resorption by osteoclasts can cause metabolic bone disorders, such as PMO.^2^, ^3^ Therefore, inhibition of osteoclast formation and/or its function may be a promising strategy for treating pathological bone loss. Although antiresorptive agents are used clinically for PMO treatment, these agents relieve clinical symptoms and serious adverse effects. For example, long‐term treatment with bisphosphonates may result in a simultaneous decrease in bone formation.^22^ Therefore, a safer agent that inhibits the progression of PMO is required. Accordingly, numerous biological compounds derived from natural products have been reported to show inhibitory effects on osteoclast differentiation and function and are emerging as new therapeutic agents with decreased side effects.^23^, ^24^, ^25^ EVO, an important alkaloidal component extracted from the fruit of Evodia rutaecarpa, has been shown to have various potent pharmacological activities. However, there is insufficient information regarding the effect on osteoclastogenesis. In this study, we demonstrated the inhibitory effect of EVO on osteoclast differentiation and functional response to RANKL stimulation. Furthermore, treatment with EVO obviously attenuated bone loss in an OVX‐induced osteoporosis mouse model.

Osteoclasts, which are monocyte‐macrophage lineage‐derived large multinucleated cells, play an important role during bone development, as they are capable of resorbing the bone matrix. As the critical cytokines for osteoclastogenesis, it is well‐known that M‐CSF induces the proliferation of early macrophage or osteoclast precursors, whereas RANKL acting via its receptor RANK induces the subsequent differentiation of osteoclast precursors into mature osteoclasts.^5^, ^6^ In this study, we found that the M‐CSF‐induced proliferation of BMMs was not affected by EVO, while EVO inhibited osteoclast function and differentiation, mainly at the early and middle stages.

When RANKL engages with RANK, it initiates a long series of downstream signalling cascades regulating osteoclast formation through the TRAF6 adaptor protein.^6^, ^26^ The NF‐κB signalling pathway modulates osteoclast differentiation as an important transcriptional factor. Stimulation triggers phosphorylation of NF‐κB p65 and IκBα, which consequently frees and translocates p65 from the cytoplasm to the nucleus, while IκBα is subsequently degraded in the cytoplasm. In the nucleus, NF‐κB p65 induces gene transcription for osteoclastogenesis by binding to specific DNA sites, such as the promoter of NFATc1. It has been reported that the deletion of p65/Rel A results in severe osteopetrosis owing to a deficiency in osteoclast formation.^19^ The activation of Ca^2+^ oscillation will also be triggered by RANKL, which leads to nuclear translocation and activates NFATc1. Negishi‐Koga et al. reported that the induction of NFATc1 is dependent on Ca^2+^‐dependent calcineurin activation.^27^ Interestingly, our study found that EVO pre‐treatment not only significantly suppressed degradation of IκBα protein, phosphorylation of p65 and the transcriptional activity of NF‐κB induced by RANKL but also down‐regulated RANKL‐induced Ca^2+^ oscillation, which contributed to the anti‐osteoclastogenic effect of EVO. c‐Fos and NFATc1 are two indispensable transcription factors in osteoclast differentiation up‐regulated by the pathways mentioned above. c‐Fos, a member of the activator protein‐1 (AP‐1) transcription factor family, is involved in the differentiation of precursor cells into bone‐resorbing osteoclasts. c‐Fos‐deficient mice exhibit osteopetrosis due to the blocking of osteoclast formation.^28^ NFATc1, an NFAT family member, has been determined to be a master executor of RANKL‐mediated osteoclast differentiation and regulates the expression of osteoclastic‐associated genes, such as TRAcP (Acp5), MMP9, CTSK and V‐ATPase d2, through cooperation with MITF and c‐Fos.^10^ In osteoclast precursors, the AP‐1 complex containing c‐Fos can trigger the auto‐amplification of NFATc1 to accelerate transcriptional processes.^29^ NFATc1‐deficient mice show the defects of impaired osteoclastogenesis, which result in the symptoms of osteopetrosis.^30^ In our study, we found that the RANKL‐induced up‐regulation of c‐Fos and NFATc1 are inhibited by EVO at the protein level. The mRNA levels of TRAcP (Acp5), c‐Fos, MMP9, CTSK are reduced by EVO in a dose‐dependent manner. Taken together, these results suggest that EVO has inhibitory effects on osteoclast differentiation and function, which significantly suppress RANKL‐induced osteoclastogenic marker expression and that the NF‐κB and calcium signalling pathways are involved in the anti‐osteoclastogenic effects of EVO.

Unlike osteoclasts, osteoblasts are responsible for bone formation and also have a main role in the mineralization of bone structures.^31^ Therefore, the effect of EVO on osteoblasts was also analysed in our study. However, no significant difference was found between the EVO and control groups. In addition, the result of the MTS assay showed that EVO was not toxic to osteoblasts at concentrations of 20 μmol/L and lower. Collectively, EVO had no effect on the differentiation and mineralization of osteoblasts.

To further investigate the effects of EVO in vivo for PMO, we established an OVX‐induced osteoporosis mouse model. The data from the micro‐CT analysis and H&E staining showed that the oral administration of EVO significantly suppressed bone loss in OVX mice. The number of activated osteoclasts around the trabecula in the evodiamine‐treated group was significantly lower than that in the OVX group. These results and those of the in vitro experiments further suggest that EVO has potential value in preventing the progression of PMO.

In conclusion, based on the in vivo and in vitro results in our study, EVO is suggested to be a safe and effective agent for treating PMO. The protective effect of EVO against PMO was accomplished by inhibiting the RANKL‐induced differentiation and function of osteoclasts and inhibiting the activation of the NF‐κB and calcium signalling pathways may be the underlying mechanism.

## CONFLICT OF INTEREST

The authors declare no conflict of interest.
