# Corrigendum

**DOI:** 10.1111/jcmm.14374

**Published:** 2019-06-20

In Jin et al^1^, the published article contains errors in Figure 2 (A), the DAPI staining of the osteoclast in the group of 5 μm EVO was previously incorrect as the image was a duplicate of RANKL group. The correct figure is shown below. The online version has been corrected.

**Figure 2:**
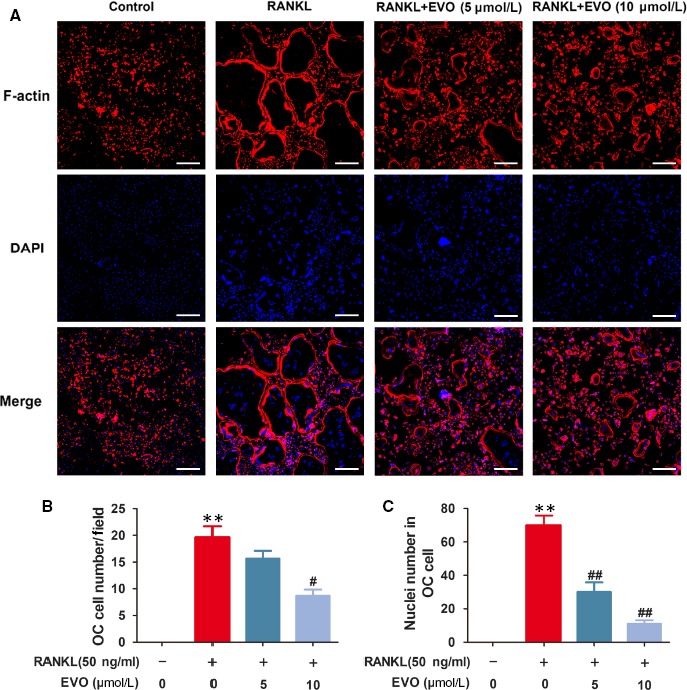
EVO inhibits RANKL‐induced F‐actin ring formation in osteoclasts. (A) The F‐actin ring formation was detected by the immunofluorescence combined with DAPI staining for nuclei. Scale bar, 200 μm. (B) Quantification of the osteoclasts treated with the indicated concentrations of EVO. (C) Average nuclei number per osteoclast under the different treatments. Data are presented as the mean ± SEM, *P < 0.05, **P < 0.01 relative to the control group. #*P* < 0.05, ##*P* < 0.01 relative to the RANKL‐induced group. n = 3

Also, the right side of figures 3, 5 and 6 was previously cropped and the correct figures are corrected in the published version.
