# Supplementary figures and images for: Evodiamine inhibits RANKL‐induced osteoclastogenesis and prevents ovariectomy‐induced bone loss in mice
Source: J Cell Mol Med. 2018 Nov 19;23(1):522–34. doi: 10.1111/jcmm.13955 (PMC6307789; doi:10.1111/jcmm.13955)

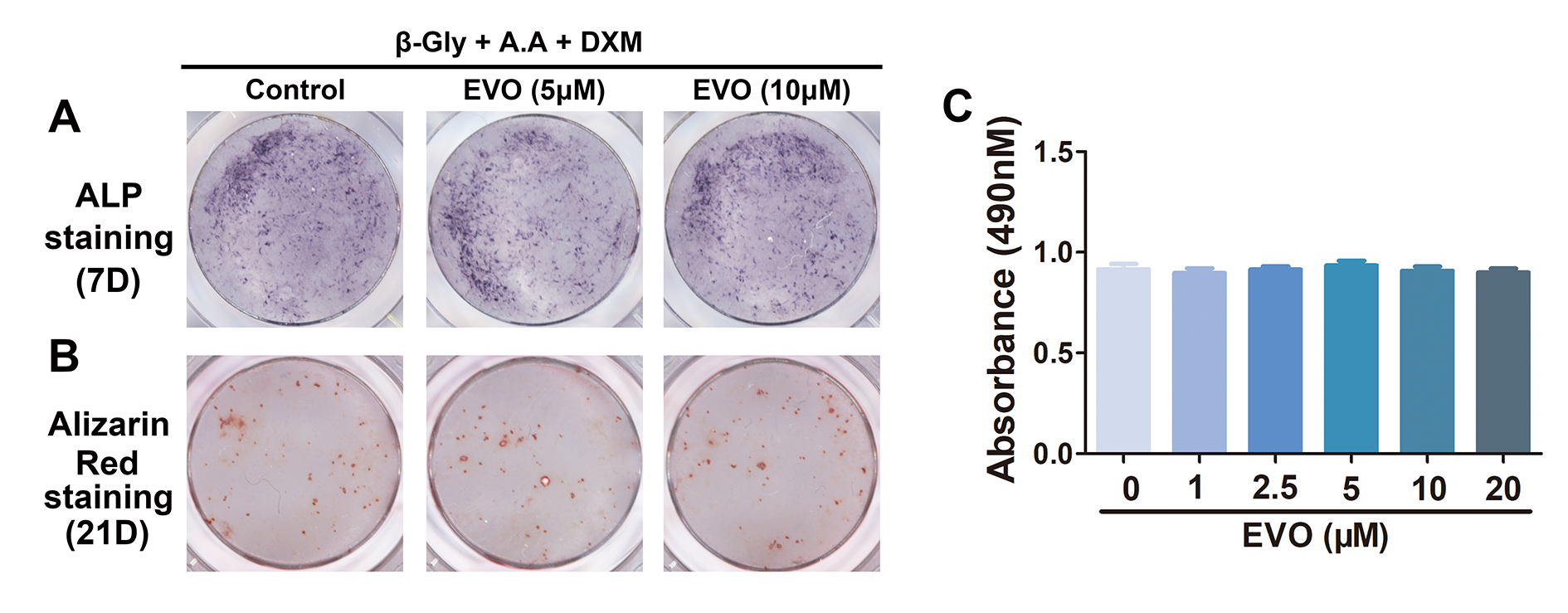

Supplement: Supplementary file 1 [file JCMM-23-522-s001.tif]
