# Supplementary material for: Evodiamine inhibits RANKL‐induced osteoclastogenesis and prevents ovariectomy‐induced bone loss in mice
Source: J Cell Mol Med. 2018 Nov 19;23(1):522–34. doi: 10.1111/jcmm.13955 (PMC6307789; doi:10.1111/jcmm.13955)
Supplement: Supplementary file 2 [file JCMM-23-522-s002.docx]

**Figure S1**: EVO did not affect the differentiation and mineralization of osteoblasts. (A) Representative images and quantitative analysis of ALP staining of primary osteoblasts in different groups (day 7). ALP-positive cells were stained blue. (B) Representative images and quantitative analysis of alizarin red staining in different groups (day 21). The calcium nodules produced by osteoblasts were stained red. (C) The effects of the indicated EVO concentrations on osteoblast viability over 48 h as measured by an MTS assay. Data are presented as the mean ± SEM, *P<0.05, **P<0.01 relative to the control group.
